# Frequency and pattern of p16 expression in neuroendocrine neoplasms

**DOI:** 10.12669/pjms.42.9.16386

**Published:** 2026-09

**Authors:** Hasan Saeed, Nadira Mamoon

**Affiliations:** 1 Dr. Hasan Saeed, MBBS, Department of Histopathology, Shifa International Hospital, Islamabad, Pakistan; 2 Dr. Nadira Mamoon, MBBS, FCPS, FRCPath (UK), Department of Histopathology, Shifa International Hospital, Islamabad, Pakistan

**Keywords:** Cell Cycle Proteins, Immunohistochemistry, Neuroendocrine Tumors, Neuroendocrine Carcinoma, p16 Protein

## Abstract

**Objectives:**

To determine the frequency and pattern of p16 expression in neuroendocrine neoplasms and evaluate its association with tumor types and grades.

**Methodology:**

This prospective descriptive study was conducted at the Department of Histopathology, Shifa International Hospital, Islamabad, from November 2024 to October 2025. A total of 85 cases of NENs were included using a non-probability consecutive sampling technique. Formalin-fixed paraffin-embedded tissue sections were evaluated using immunohistochemistry for p16 expression. Clinicopathological variables including age, gender, tumor site, subtype, and grade were recorded. Data were analyzed using SPSS version 27.0, and associations were assessed using the chi-square test, with p<0.05 considered statistically significant.

**Results:**

The mean age was 55.8±14.66 years, with male predominance in 56.5% (48) cases. Well-differentiated neuroendocrine tumors comprised 69.4% (59) of cases, while neuroendocrine carcinomas accounted for 30.6% (26). The liver was the most common site in 29.4% (25) cases. Diffuse p16 positivity was observed in 24.7% (21) cases, heterogeneous positivity in 32.9% (28), and negative staining in 42.4% (36). Diffuse p16 expression was significantly more frequent in neuroendocrine carcinomas than in well-differentiated neuroendocrine tumors [65.4% (17) vs. 6.8% (4), p < 0.001], with an odds ratio of 25.97 (95% CI: 7.10–95.05).

**Conclusion:**

Diffuse p16 expression was significantly more frequent in neuroendocrine carcinomas than in well-differentiated neuroendocrine tumors, while heterogeneous and negative p16 expression predominated in well-differentiated tumors. p16 immunohistochemistry may serve as a useful adjunct in distinguishing neuroendocrine carcinomas from Grade-3 well-differentiated neuroendocrine tumors in diagnostically challenging cases.

## INTRODUCTION

Neuroendocrine neoplasms (NENs) represent a diverse group of epithelial tumors originating from neuroendocrine cells widely distributed throughout the human body, particularly within the gastrointestinal and respiratory tracts. Although relatively uncommon, their incidence has increased substantially over recent decades, with considerable variation according to geographic region, primary tumor site, classification criteria, and methods of case ascertainment.^1^ An apparent rise in incidence has been observed over recent decades, largely attributed to improved diagnostic awareness, advances in imaging techniques, and increasing utilization of immunohistochemistry. Local epidemiological data from Pakistan remain limited; however, a single-center study from Shifa International Hospital, Islamabad, reported the pancreas as the most frequent primary site among gastroenteropancreatic neuroendocrine tumors (28%).^2^ According to the WHO Classification of Tumours, 5^th^ edition, epithelial neuroendocrine neoplasms are broadly classified into well-differentiated neuroendocrine tumors (NETs) and poorly differentiated neuroendocrine carcinomas (NECs).^3^ Well-differentiated NETs typically demonstrate characteristic organoid architectural patterns such as trabecular, nested, or glandular arrangements, relatively uniform cytological features, limited necrosis, and comparatively favorable clinical outcomes. In contrast, NECs are composed of poorly differentiated small or large cells, frequently exhibit extensive necrosis and high proliferative activity, aggressive clinical behavior, and poor survival outcomes.

Grading of well-differentiated NETs is primarily determined by proliferative activity assessed using mitotic count and Ki-67 labeling index, categorizing tumors into Grade-1 (G1), Grade-2 (G2), and Grade-3 (G3). Recognition of G3 NETs has introduced a significant diagnostic challenge, as these tumors may demonstrate proliferative indices overlapping with NECs. Current classification systems therefore recommend distinguishing G3 NETs from NECs, as NECs are inherently high-grade neoplasms and are no longer assigned numerical grades to minimize diagnostic ambiguity. Treatment recommendations for NETs and NECs also differ.^3^,^4^ p16 is a cyclin-dependent kinase inhibitor encoded by the CDKN2A tumor suppressor gene located on chromosome 9p21. It regulates cell cycle progression by inhibiting CDK4/CDK6-mediated phosphorylation of the retinoblastoma (RB) protein, thereby preventing transition from the G1 to S phase. Disruption of the RB pathway results in compensatory overexpression of p16 protein.^5^-^7^ Recent studies have demonstrated that p16 immunoexpression differs between well-differentiated NETs and NECs. Umetsu et al. reported integrated genomic and clinicopathological differences between pancreatic Grade-3 NETs and NECs, highlighting distinct molecular alterations and differential p16 expression patterns.^8^ Similarly, Papaxoinis et al. demonstrated variable expression of RB1, cyclin D1, and p16 in pulmonary small cell and large cell neuroendocrine carcinomas, supporting the diagnostic utility of these markers in high-grade neuroendocrine neoplasms.^9^ However, the frequency and pattern of p16 expression, particularly in Grade-3 NETs compared with NECs, remain insufficiently characterized, with limited data available from Pakistan.

The present study aimed to determine the frequency and pattern of p16 expression in NENs using immunohistochemical (IHC) analysis and to evaluate its distribution across tumor grades and subtypes, with particular emphasis on the comparison between Grade-3 NETs and NECs.

## METHODOLOGY

This prospective descriptive study was conducted at the Department of Histopathology, Shifa International Hospital, Islamabad, a tertiary care facility receiving specimens from nationwide collection centers, from November 2024 to October 2025. NEN cases were prospectively recruited consecutively as they were received during the study period. Relevant clinical and pathological data were recorded prospectively using a structured data collection proforma. p16 IHC analysis was performed on formalin-fixed, paraffin-embedded tumor tissue obtained from specimens submitted for routine diagnostic evaluation during the study period.

### Ethical approval:

Ethical approval was obtained from the Institutional Review Board of Shifa International Hospital, Islamabad (IRB No. 145-24, dated 4th April 2024). Patient confidentiality was maintained.

As IHC analysis was performed on formalin-fixed, paraffin-embedded tissue specimens submitted for routine diagnostic purposes during the study period, the requirement for individual informed consent was waived by the ethical committee.

The sample size was calculated using the single-population proportion formula, based on an expected proportion of 69% derived from the study by Umetsu et al.^8^, which reported diffuse strong p16 expression in 69% of pancreatic NECs. Assuming a 95% confidence level and a 10% margin of error, the calculated minimum sample size was 83 cases. The calculation was performed as follows:

n = Z^2^P(1−P)/d^2^

where; **n =** required sample size, **Z =** 1.96 for a 95% confidence level, **P =** expected proportion (0.69),

**d =** margin of error (0.10).

n=(1.96)^2^(0.69)(1−0.69)/(0.10)^2^ = 82.56

Thus, the minimum required sample size was rounded up to 83 cases, and a total of 85 eligible cases were enrolled. All consecutive eligible NEN cases received during the study period were enrolled using a non-probability consecutive sampling technique.

### Inclusion and Exclusion criteria:

All consecutive eligible cases diagnosed as NENs on histopathological examination during the study period were included, comprising well-differentiated neuroendocrine tumors (NETs; G1, G2, and G3) and neuroendocrine carcinomas (NECs; small cell and large cell types). Both biopsy and resection specimens were included. Recurrent and previously treated tumors were excluded. Primary and metastatic tumors were not distinguished and were included if they fulfilled the diagnostic criteria for NENs. Multiple specimens from the same patient were not included, with each patient contributing one case to the analysis. Mixed neuroendocrine–non-neuroendocrine neoplasms (MiNENs), poorly fixed specimens, and cases with insufficient tumor tissue for IHC evaluation were excluded. The unit of analysis was the individual patient/case. NENs were classified as well-differentiated NETs or NECs (small cell or large cell type) according to the WHO Classification of Tumours criteria, based on histomorphological features and proliferative activity. NETs were further classified as G1, G2, or G3 according to WHO criteria.

Formalin-fixed specimens were grossed according to standard laboratory protocols and embedded in paraffin. Sections measuring 3–5-µm were prepared and stained with hematoxylin and eosin (H&E) for routine histopathological examination. Representative tumor blocks were selected for IHC analysis. IHC for p16 was performed on 4-µm sections obtained from formalin-fixed, paraffin-embedded tissue blocks using the Benchmark XT automated slide stainer (Ventana Medical Systems, Tucson, AZ, USA). Sections were deparaffinized and subjected to antigen retrieval using Cell Conditioning Solution (high pH CC1 standard) for 60 minutes. Slides were incubated for 44 minutes with mouse monoclonal anti-p16 antibody (clone E6H4™, CINtec® p16 Histology, dilution 1:100), followed by detection and amplification according to the manufacturer’s instructions. Appropriate positive tissue controls were included with each IHC run. The positive control tissue was selected based on established p16 expression and included predominantly high grade serous carcinoma of ovary. All cases were evaluated independently by the principal investigator, and findings were verified by a consultant histopathologist. Both evaluators were aware that the cases represented neuroendocrine neoplasms but were blinded to the specific histopathological diagnosis and tumor grade during assessment of p16 expression.

Operational definitions were established for interpretation of p16 IHC expression. Diffuse positive expression was defined as strong combined nuclear and cytoplasmic staining in >70% of tumor cells. Heterogeneous positive expression was defined as patchy or variable combined nuclear and cytoplasmic staining in 10–70% of tumor cells. Negative expression was defined as absent or focal combined nuclear and cytoplasmic staining in <10% of tumor cells. These operational thresholds were author-derived for the purpose of this study and were applied consistently across all cases. Relevant clinical and pathological data were recorded using a structured data collection proforma. Variables included patient age, gender, anatomical site of the tumor, histological type (NET or NEC), tumor grade, and pattern of p16 expression.

### Statistical analysis:

Data were analyzed using SPSS version 27.0. Quantitative variables such as age were summarized as mean ± standard deviation. For analysis of its association with p16 expression, age was categorized into <40 and ≥40 years. Categorical variables were presented as frequencies and percentages, and associations between categorical variables were assessed using the Chi-square test. A p-value of <0.05 was considered statistically significant. Missing data were excluded from the respective analysis.

## RESULTS

A total of 85 cases of NENs were included in the study. Amongst these, 69.4% (59) were NETs and 30.6% (26) were NECs. The mean age of the patients was 55.87 ± 14.66 years (range: 11–86 years). Most patients were older than 40 years, 87.1% (74), while 12.9% (11) were younger than 40 years. The mean age of patients with NETs was 51.39±13.70 years (range: 11–84 years), whereas patients with NECs had a higher mean age of 66.04±11.47 years (range: 42–86 years) (Table-I). Although NECs tended to occur in relatively older patients than well-differentiated NETS, this association was not statistically significant (χ² = 3.116, df = 1, p = 0.078). There was a slight male predominance with 56.5% (48) males and 43.5% (37) females.

**Table-I:** Clinical and pathological characteristics of the study population (n = 85), presented as percentage followed by frequency in parentheses.

Variable	Category	Frequency
Age (years)	Mean ± SD	55.87 ± 14.66
	Range	11–86
	<40 years	12.9% (11)
	≥40 years	87.1% (74)
Gender	Male	56.5% (48)
	Female	43.5% (37)
Anatomical site of lesion	Liver	29.4% (25)
	Pancreas	12.9% (11)
	Stomach	8.2% (7)
	Bronchus	5.9% (5)
	Duodenum	5.9% (5)
	Appendix	4.7% (4)
	Lymph node	4.7% (4)
	Colon	3.5% (3)
	Lung	3.5% (3)
	Small bowel	3.5% (3)
	Cervical lymph node	2.4% (2)
	Esophagus	2.4% (2)
	Adrenal gland	1.2% (1)
	Ampulla	1.2% (1)
	Gastroesophageal junction	1.2% (1)
	Kidney	1.2% (1)
	Mediastinum	1.2% (1)
	Mesentery	1.2% (1)
	Paraspinal	1.2% (1)
	Rectum	1.2% (1)
	Spinal	1.2% (1)
	Urinary bladder	1.2% (1)
	Vertebra	1.2% (1)
Histological classification	Neuroendocrine tumor (NET)	69.4% (59)
	Grade-1 NET	42.4% (25)*
	Grade-2 NET	45.8% (27)*
	Grade-3 NET	11.9% (7)*
	Neuroendocrine carcinoma (NEC)	30.6% (26)
	Small cell carcinoma	92.3% (24)†
	Large cell NEC	7.7% (2)†

* Percentages for NET grades are calculated using NETs as the denominator (n = 59); † Percentages for NEC subtypes are calculated using NECs as the denominator (n = 26).

The liver was the most common site of the specimens evaluated, accounting for 29.4% (25) cases, followed by the pancreas in 12.9% (11) and stomach in 8.2% (7) cases. Other sites included bronchi and duodenum, in 5.9% (5) cases each, appendix and lymph node, in 4.7% (4) cases each, and colon and lung, in 3.5% (3) cases each. The anatomical site refers to the site of the submitted specimen and was not categorized as primary or metastatic in this study. Among NETs, Grade-2 tumors were most frequent making up 31.8% (27) cases, followed by Grade-1 in 29.4% (25) cases and Grade-3 in 8.2% (7) cases. Small cell carcinoma represented 28.2% (24) of total cases, whereas large cell neuroendocrine carcinoma accounted for 2.4% (2) (Table-I).

Analysis of p16 expression demonstrated that diffuse positivity was most prominent in 66.7% (16) small cell carcinoma cases followed by large cell NEC in 50.0% (1) cases. Among NETs, diffuse expression was seen in 8.0% (2) Grade-1, 3.7% (1) Grade-2 and 14.3% (1) Grade-3. Respectively, no staining was observed in 60.0% (15) of Grade-1, 40.7% (11) of Grade-2 and 57.1% (4) of Grade-3 NETs (Fig.1) (Table-II).

**Fig.1:**
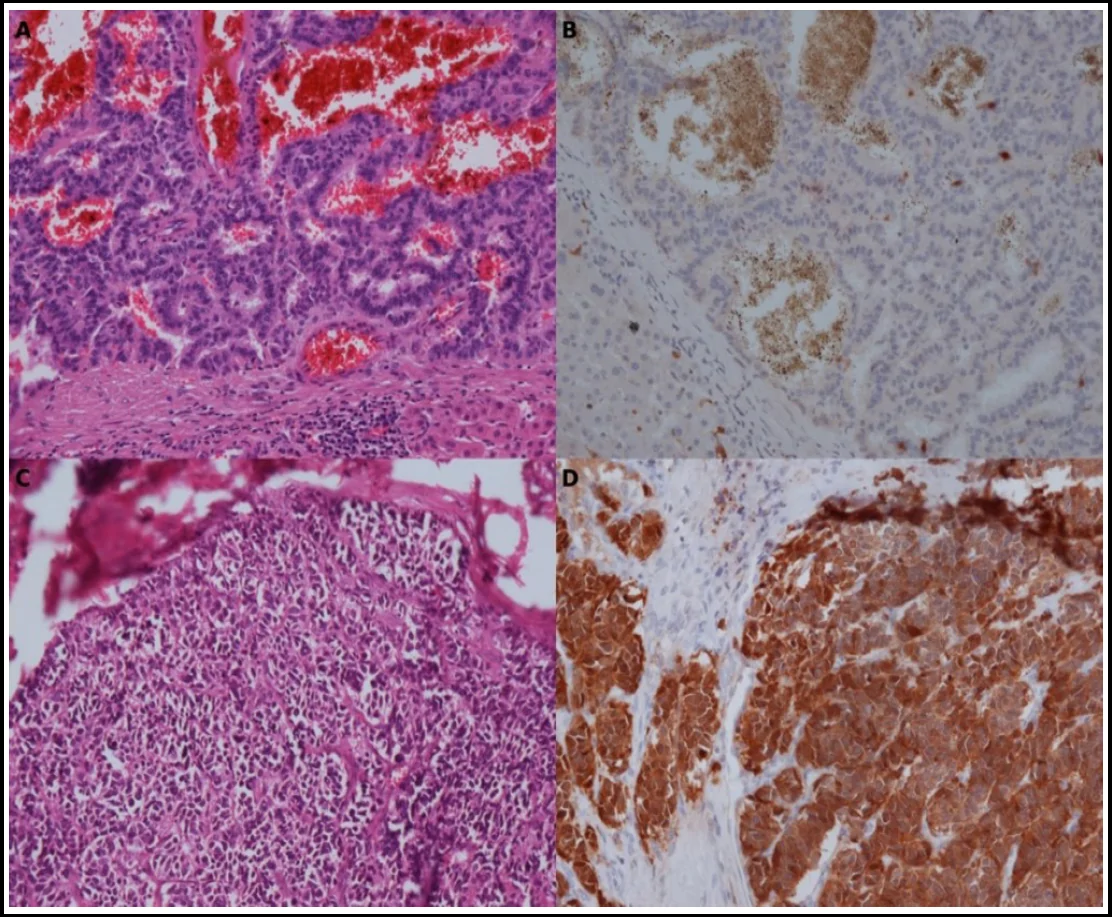
Photomicrographs demonstrating neuroendocrine tumors and p16 immunoexpression (x 200 magnification). (A) Grade-1 neuroendocrine tumor showing typical organoid architecture on H&E staining. (B) Corresponding tumor showing negative p16 immunostaining. (C) Small cell carcinoma composed of nests of small cells with hyperchromatic nuclei and scant cytoplasm on H&E. (D) Small cell carcinoma demonstrating diffuse block positivity for p16.

**Table-II:** Distribution of p16 expression according to tumor type and histological subtype in neuroendocrine neoplasms (n = 85).

Tumor type / subtype	Diffuse	Heterogeneous	Negative	Total
Neuroendocrine tumors (NETs)	6.8% (4)	42.4% (25)	50.8% (30)	59
Grade-1 NET	8.0% (2)	32.0% (8)	60.0% (15)	25
Grade-2 NET	3.7% (1)	55.6% (15)	40.7% (11)	27
Grade-3 NET	14.3% (1)	28.6% (2)	57.1% (4)	7
Neuroendocrine carcinomas (NECs)	65.4% (17)	11.5% (3)	23.1% (6)	26
Small cell carcinoma	66.7% (16)	12.5% (3)	20.8% (5)	24
Large cell NEC	50.0% (1)	0% (0)	50.0% (1)	2

Diffuse p16 expression was significantly more frequent in NECs than in well-differentiated NETs [65.4% (17) vs. 6.8% (4), p < 0.001], with an odds ratio of 25.97 (95% CI: 7.10–95.05).

p16 expression was also assessed in relation to demographic variables. A statistically significant association was observed between age group and p16 expression (χ² = 6.843, df = 2, p = 0.033). Diffuse p16 expression was observed in 28.4% (21) of patients aged ≥40 years and in 0% (0) of patients aged <40 years. Heterogeneous p16 expression was observed in 28.4% (21) and 63.6% (7) of patients aged ≥40 and <40 years, respectively. No statistically significant association was observed between gender and p16 expression (χ² = 3.072, df = 2, p = 0.215); diffuse p16 expression was observed in 31.3% (15) of males and 16.2% (6) of females.

## DISCUSSION

NENs represent a heterogeneous group of tumors characterized by variable differentiation, biological behavior, and clinical outcomes. Accurate distinction between well-differentiated NETs and NECs is essential because these entities differ substantially in prognosis and therapeutic management. Immunohistochemical markers involved in cell-cycle regulation have therefore been increasingly explored as adjunct tools in the diagnostic evaluation of NENs, particularly in limited biopsy specimens.^10^

In the present study, the mean age of patients was 55.87 ± 14.66 years, and the majority of cases occurred in individuals older than 40 years, 87.1% (74). Similar demographic patterns have been reported in several studies evaluating NENs. Ribeiro and Maleki observed that these neoplasms predominantly affect middle-aged and older adults.^11^ NECs in the present cohort tended to occur at older ages than well-differentiated NETs, although this trend did not reach statistical significance. A slight male predominance was observed in our cohort. Comparable gender distributions have been reported in previous clinicopathological series of neuroendocrine neoplasms.^12^ Bahra et al. reported a similar male predominance in their study of NECs of the head and neck.^13^

With regard to tumor subtype distribution, well-differentiated NETs constituted the majority of cases in our study, 69.4% (59), whereas NECs accounted for 30.6% (26) cases. These findings are consistent with previously published data indicating that well-differentiated NETs are more common than poorly differentiated NECs in clinical practice, as described in the WHO Classification of Tumours.^14^,^15^ Inzani et al. highlighted the relative rarity but aggressive nature of NECs in their clinicopathological study of neuroendocrine carcinoma of the uterine cervix.^14^

In the present study, the liver represented the most common site of tumor involvement in 29.4% (25), followed by pancreas in 12.9% (11) and stomach in 8.2% (7) cases. The predominance of liver involvement may reflect the well-recognized tendency of neuroendocrine tumors to metastasize to the liver. Manneh-Kopp et al. similarly emphasized that hepatic involvement is frequently encountered in patients with neuroendocrine neoplasms.^15^

Immunohistochemical evaluation of p16 expression demonstrated diffuse positivity in 24.7% (21), heterogeneous positivity in 32.9% (28), and negative staining in 42.4% (36) cases. The p16 protein functions as a key regulator of the G1–S transition of the cell cycle by inhibiting cyclin-dependent kinases. Alterations in the p16–RB pathway have been implicated in the pathogenesis of several malignancies and are frequently associated with highly proliferative tumors. Forsythe et al. highlighted the importance of disruptions in cell-cycle regulatory pathways in the development and progression of NETs.^16^ The most significant finding of the present study was the strong association between diffuse p16 expression and NECs. Diffuse positivity was observed in 65.4% (17) of NECs compared with only 6.8% (4) of NETs. These results support previous observations indicating that p16 overexpression is frequently associated with NECs.

In pulmonary NECs, Švajdler et al. demonstrated that p16 immunohistochemistry may serve as a useful supplementary marker in small biopsies and cytology specimens where morphological evaluation is limited.^10^ Likewise, Ribeiro and Maleki reported that p16 immunostaining can assist in improving diagnostic accuracy in cytological samples.^11^ Molecular studies further support these findings.

The differing patterns of p16 immunoexpression observed in this study reflect alterations in the p16–RB cell-cycle regulatory pathway. Diffuse p16 expression is commonly associated with RB pathway inactivation, whereas well-differentiated neuroendocrine tumors typically retain RB pathway integrity and therefore show absent or heterogeneous staining. Variability in staining distribution may also reflect intratumoral molecular heterogeneity.^17^,^18^ Yachida et al. demonstrated that NECs frequently harbor genetic alterations involving TP53 and RB1, leading to dysregulation of the p16–RB pathway.^19^

Subtype analysis in our study revealed that diffuse p16 expression was most frequently observed in small-cell carcinomas, 66.7% (16). In contrast, well-differentiated NETs predominantly demonstrated heterogeneous or negative staining patterns. Liu S et al. similarly demonstrated variable p16 expression in gastroenteropancreatic NETs.^20^ De Wispelaere et al. also reported a high prevalence of p16 staining in malignant tumors with aggressive biological behavior.^21^

The distinction between well-differentiated G3 NETs and poorly differentiated NECs represents one of the most challenging areas in neuroendocrine pathology. These entities may demonstrate overlapping proliferative indices, particularly in terms of Ki-67 labeling index and mitotic activity, making morphological differentiation difficult in limited biopsy specimens. In such scenarios, ancillary immunohistochemical markers reflecting alterations in cell-cycle regulatory pathways may provide additional diagnostic information. Diffuse p16 expression observed predominantly in neuroendocrine carcinomas in the present study supports the potential utility of this marker in resolving diagnostically challenging cases where G3 NETs and NECs may show overlapping proliferative features.^19^

In addition to tumor subtype, our study demonstrated a statistically significant association between age and p16 expression (p = 0.033). Joseph et al. demonstrated that the progression of low-grade NETs to high-grade carcinomas may be associated with accumulating genetic alterations over time, which may partly explain the age-related patterns observed in our study.^22^ No statistically significant association was identified between gender and p16 expression (p = 0.215). Diffuse positivity was observed in 31.3% (15) of male patients compared with 16.2% (6) of female patients.

### Limitations

This study was conducted at a single tertiary care institution with a relatively limited sample size, which may affect the generalizability of the findings. Additionally, certain tumor subtypes were represented by small numbers, particularly Grade-3 NETs (n = 7), limiting the statistical power of subgroup analyses, including comparisons between Grade-3 NETs and NECs. The lack of clinical follow-up data precluded assessment of the prognostic significance of p16 expression. Additionally, the p16 expression thresholds used in this study were author-derived and have not been independently validated.

### Recommendations

Incorporation of p16 into routine diagnostic panels may enhance diagnostic accuracy, especially in limited biopsy samples where morphology alone may be insufficient. Furthermore, in resource-limited settings, p16 may act as a cost-effective surrogate marker to aid in tumor stratification, potentially reducing reliance on extensive IHC panels. Future multicenter studies with larger sample sizes and clinical follow-up are recommended to further validate the diagnostic and prognostic utility of p16 in NENs.

## CONCLUSION

Diffuse p16 expression demonstrates a strong association with NECs compared with NETs and can serve as a useful diagnostic adjunct, particularly in small biopsy specimens. Its application may aid in accurate tumor classification and grading, especially in morphologically challenging cases. However, larger studies with clinical follow-up are required to further establish its prognostic significance and validate its routine use.
